# Evaluation of Chinese healthcare organizations' innovative performance in the digital health era

**DOI:** 10.3389/fpubh.2023.1141757

**Published:** 2023-07-06

**Authors:** Wenjun Gu, Luchengchen Shu, Wanning Chen, Jinhua Wang, Dingfeng Wu, Zisheng Ai, Jiyu Li

**Affiliations:** ^1^School of Medicine, Tongji University, Shanghai, China; ^2^Institute of Neuroscience, Center for Excellence in Brain Science and Intelligence Technology, Chinese Academy of Science, Shanghai, China; ^3^School of Life Sciences and Technology, Tongji University, Shanghai, China; ^4^Shanghai Institute of Medical Innovation Technology Transfer, Shanghai, China; ^5^National Clinical Research Center for Child Health, Children's Hospital, Zhejiang University School of Medicine, Hangzhou, China; ^6^Huadong Hospital, Fudan University, Shanghai, China

**Keywords:** innovation performance, evaluation model, Science-technology relevance, Chinese hospitals, digital health era, factor analyses (FA), analytical hierarchy process (AHP), logistic regression (LG)

## Abstract

**Background:**

Healthcare workers' relationship with industry is not merely an agent mediating between consumer and vendor, but they are also inventors of the interventions they exist to deliver. Driven by the background of the digital health era, scientific research and technological (Sci-tech) innovation in the medical field are becoming more and more closely integrated. However, scholars shed little light on Sci-tech relevance to evaluate the innovation performance of healthcare organizations, a distinctive feature of healthcare organizations' innovation in the digital health era.

**Methods:**

Academic publications and patents are the manifestations of scientific research outputs and technological innovation outcomes, respectively. The study extracted data from publications and patents of 159 hospitals in China to evaluate their innovation performance. A total of 18 indicators were constructed, four of which were based on text similarity match and represented the Sci-tech relevance. We then applied factor analyses, analytical hierarchy process, and logistic regression to construct an evaluation model. We also examined the relationship between hospitals' innovation performance and their geographical locations. Finally, we implemented a mediation analysis to show the influence of digital health on hospital innovation performance.

**Results:**

A total of 16 indicators were involved, four of which represented the Sci-tech including the number of articles matched per patent (NAMP), the number of patents matched per article (NPMA), the proportion of highly matched patents (HMP), and the proportion of highly matched articles (HMA). Indicators of HMP (*r* = 0.52, *P* = 2.40 × 10^−12^), NAMP (*r* = 0.52, *P* = 2.54 × 10^−12^), and NPMA (*r* = 0.51, *P* = 5.53 × 10^−12^) showed a strong positive correlation with hospital innovation performance score. The evaluation model in this study was different from other Chinese existing hospital ranking systems. The regional innovation performance index (RIP) of healthcare organizations is highly correlated with per capita disposable income (*r* = 0.58) and regional GDP (*r* = 0.60). There was a positive correlation between digital health innovation performance scores and overall hospital innovation performance scores (*r* = 0.20). In addition, the hospitals' digital health innovation performance affected the hospital's overall innovation score with the mediation of Sci-tech relevance indicators (NPMA and HMA). The hospitals' digital health innovation performance score showed a significant correlation with the number of healthcare workers (*r* = 0.44).

**Conclusion:**

This study constructed an assessment model with four invented indicators focusing on Sci-tech relevance to provide a novel tool for researchers to evaluate the innovation performance of healthcare organizations in the digital health era. The regions with high RIP were concentrated on the eastern coastal areas with a higher level of economic development. Therefore, the promotion of scientific and technological innovation policies could be carried out in advance in areas with better economic development. The innovations in the digital health field by healthcare workers enhance the Sci-tech relevance in hospitals and boost their innovation performance. The development of digital health in hospitals depends on the input of medical personnel.

## 1. Introduction

Healthcare does not merely administer the market for interventions, it determines—professionally, not commercially—both their value and much of the biological basis for their development (1). Healthcare workers' relationship with industry is not merely an agent mediating between consumer and vendor, but they are also inventors of the interventions they exist to deliver. The digital health era accelerates collaboration between medical professionals and the industry. The United States Food and Drug Administration (FDA) approved the reSet of Pear Therapeutics in September 2017. The guidelines of the World Health Organization (WHO) on tuberculosis (TB) treatment provided the first-ever WHO evidence-based recommendations on the use of phones, video, or electronic medication monitors to help patients adhere to TB medication and deliver TB care (1). A variety of DTx products are currently available for managing diabetes (2, 3), treating patients with social anxiety disorder (4), neurological disorders (5), and mental illness (6), and developing digital biomarkers to predict treatment response (7). Shanghai United Imaging Medical Technology Co., Ltd., in collaboration with Zhongshan Hospital, launched the Time-of-Flight Intracranial MRA at 5T in 2022. Driven by the background of the digital health era, scientific research and technological (Sci-tech) innovation in the medical field are becoming more and more closely integrated. The advent of the digital health era is changing the innovative behavior of hospitals.

The hospital evaluation system has a guiding effect on innovation in medical institutions. Thus, many countries around the world are carrying out research to develop healthcare evaluation systems, such as the U.S. News & World Report's “America's Best Hospitals” (8), “British Health Care Quality Assessment System” (8), “Truven Health 100 Top Hospitals” (9), and “Healthgrades Best Hospitals” (9). These ranking systems are different in the selection of indicators and methods to construct their models, as well as in their emphasis on hospital quality. However, they all mainly focus on indicators, such as survival rate, infectious rate, and customer satisfaction, reflecting outcomes or outputs of medical services and the scale and operation of hospitals.

With the development of the biomedical industry, the innovative behavior of hospitals is receiving more and more attention from academics, and hospital evaluation systems focusing on innovation performance have emerged in China. Our group systematically reviewed four major hospital ranking systems in China, including Chinese hospital competitiveness rankings, Chinese hospital science and technology value rankings, Chinese best hospital rankings, and Chinese hospital Natural Index rankings, and found that the quality and quantity of SCI publications, the key indicators of national projects, and top academic talents were the most frequent factors used to evaluate the level of hospital scientific research (10).

Scientific activities have increasingly played an important role in industrial innovation, and more firms are relying on external sources of scientific knowledge generated mainly by medical universities and hospitals. A large number of efforts on theory and model exploration as well as on empirical studies have been extensively undertaken to uncover the nature, mechanism, directionality, and magnitude of the transfer of that knowledge between science and technology (11). Science-technology innovation linkage analysis has been implemented in the field of pharmaceutical innovation (12), medical and laboratory equipment (13), and biomedical innovation (14).

Understanding the complex relationship between science and technology has been becoming more important than ever before for innovation-related studies. However, scholars shed little light on Sci-tech relevance when they evaluate the innovation performance of hospitals, a distinctive feature of healthcare organizations' innovation in the digital health era.

Academic publications and patents are the manifestations of scientific research outputs and technological innovation outcomes, respectively (15). As healthcare moves into the digital age, the value of mutual reference and support between publications and patents has become increasingly prominent. Due to publications and patents being isolated from each other and the lack of cross-referencing in the current document service system (16), existing evaluation systems of hospital innovation performance still exist in the stage of simply counting the number of publications and granted patents. It is difficult to update the existing evaluation system of medical institutions to pay much attention to the relevance of Sci-tech innovations. In the long term, this will hinder the innovation performance of healthcare organizations. Fortunately, with the advancement of machine learning, text similarity algorithms can match documents based on the appearance of the same or similar words (17). In this study, we constructed an original evaluation model for Chinese hospitals related to innovation performance. The new model included not only traditional indicators such as the quality and quantity of SCI publications and the number of authorized patents in the existing evaluation system but also five novel indicators to emphasize the relevance between science and technology.

## 2. Methods

### 2.1. Data collection

Hospitals included in this study were selected from the top 100 hospitals of four representative Chinese hospital ranking systems (Chinese hospital competitiveness rankings, Chinese hospital science and technology value rankings, Chinese best hospital rankings, and Chinese hospital Natural Index rankings), and a total of 164 unique hospitals were obtained (Table 1). Five purely medical research institutions, the Institute of Development and Regenerative Biology, Beijing Institute for Brain Disorders, MOE Key Laboratory of Molecular Cardiovascular Science, The Fourth School of Clinical Medicine of NJMU, and Key Laboratory of Assisted Reproduction of Peking University, were excluded (Supplementary Table S1). Through the search queries (Supplementary Table S1), 692,342 articles published by 159 hospitals were retrieved from the Web of Science (https://www.webofscience.com) and 45,106 patents were retrieved from incoPat, a patent database provider from China with a collection of patents from 120 authorities (https://www.incopat.com). In addition, several regional development characteristics were obtained from the China National Bureau of Statistics (https://www.stats.gov.cn) for further comparative analysis (Table 1 and Supplementary Table S2).

**Table 1:** Data and data sources.

**Data resources**	**Data contents**	**Data volumes**
Chinese hospitals' competitiveness rankings (https://rank.cn-healthcare.com/)	Top 100 hospitals	100 hospitals
Chinese hospital science and technology evaluation metrics (https://www.pumc.edu.cn/cms/web/search/index.jsp)	Top 100 hospitals	100 hospitals
China's hospital rankings (https://www.ailibi-gaha.com/login)	Top 100 hospitals	100 hospitals
Nature index (https://www.springernature.com/cn)	Top 100 hospitals	100 hospitals
Official websites of hospitals	Hospital names and addresses	164 hospitals
Web of science (https://www.webofscience.com/wos/)	Hospital articles during 2000–2019	6,92,342 articles
incoPat (https://www.incopat.com/)	Hospital patents during 2000–2019	45,106 patents
China national bureau of statistics (http://www.stats.gov.cn/)	Regional GDP of 2019 (100 million yuan)^*^	31 regions
	Local finical healthcare expenditure in 2018 (100 million yuan)^*^	31 regions
	Per capita disposable income in 2019 (yuan)^*^	31 regions
	The number of hospitals in 2018^*^	31 regions
	The number of healthcare workers in 2018 (10,000 persons)^*^	31 regions
	Resident population at the end of 2019 (10,000 persons)^*^	31 regions

* The data from Hong Kong, Macao, and Taiwan are not included. GDP: gross domestic product.

Based on the academic publications and patents of hospitals, we designed 18 hospital indicators, including four article indicators, nine patent indicators, and five article-patent relevance indicators (Table 2). Among them, NPMA, NAMP, HMP, HMA, and PAR were specifically used to characterize the cross-referencing between publications and patents, and the other indicators were from existing ranking systems of hospitals or studies (Table 2). We organized a team of experts to discuss the reasonableness, science and feasibility of the indicators, including one patent lawyer specializing in healthcare technologies, two experts in bioinformatics, an expert in epidemiological statistics, and a physician who majored in artificial intelligence in healthcare.

**Table 2:** Eighteen hospital innovation performance indicators based on publications and patents.

**Indicators**	**Abb**.	**Explanation**	**Source**	**Mean**	**Min**	**Max**
**Article indicators**						
Proportion of highly cited articles	PHCA	Number of highly cited papers^#^/NA	Chinese hospital science and technology evaluation metrics	5.5 × 10^−3^	0.0	2.2 × 10^−2^
Proportion of hot articles	PHA	Number of hot papers^*^/NA	Chinese hospital science and technology evaluation metrics	5.0 × 10^−4^	0.0	7.1 × 10^−3^
Increasing rate of articles	AI	Article increasement per year/NA	Li Shiji and Shikai (18)	1.6 × 10^−2^	−7.7 × 10^3^	4.7 × 10^−2^
**Patent indicators**						
Number of patents	NP	Number of granted patents during 2000–2019	World intellectual property indicator (https://www.wipo.int/publications/zh/series/index.jsp?id=37)	2.8 × 10^2^	0.0	2.5 × 10^3^
Proportion of applied invention patents	PAIP	Number of applied invention patents/NP	World intellectual property indicators	8.1 × 10^−1^	0.0	4.0
Proportion of granted invention patents	PGIP	Number of granted invention patents/NP	China hospital innovation transformation ranking (https://innovation-rank.cn-healthcare.com/)	2.3 × 10^−1^	0.0	9.9 × 10^−1^
Proportion of utility model patents	PUP	Number of utility model patents/NP	World intellectual property indicators	7.2 × 10^−1^	0.0	1.0
Proportion of design patents	PDP	Number of design patents/NP	World intellectual property indicators	3.0 × 10^−2^	0.0	4.0 × 10^−1^
Cited number per patent family	CNP	Cited number of patent family/NP	Sun et al. (19)	1.9	0.0	4.0 × 10^1^
Increasing rate of patents	PI	Patent increasement per year/NP	Li Shiji and Shikai (18)	5.2 × 10^−2^	−8.3 × 10^2^	2.0 × 10^−1^
Ratio of patent transferring	PTR	Number of patent transferring/NP	China hospital innovation transformation ranking	6.3 × 10^−2^	0.0	9.9 × 10^−1^
Ratio of patent licensing	PLR	Number of patent licensing/NP	China hospital innovation transformation ranking	2.9 × 10^−3^	0.0	7.5 × 10^−2^
**Article-patent relevance indicators**						
Patent-article ratio	PAR	NP/NA	Etzkowitz and Leydesdorff (11)	8.7 × 10^−2^	0.0	8.3 × 10^−1^
Number of patents matched per article	NPMA	Number of article-patent matches/NA	Etzkowitz and Leydesdorff (11)	1.5 × 10^−2^	0.0	9.0 × 10^−2^
Number of articles matched per patent	NAMP	Number of article-patent matches/NP	Inspired by Etzkowitz and Leydesdorff (11)	4.2 × 10^−1^	0.0	1.8 × 10^1^
Proportion of high-matched patents	HMP	Number of high-matched patents/NP	Inspired by Etzkowitz and Leydesdorff (11)	5.4 × 10^−2^	0.0	4.3 × 10^−1^
Proportion of high-matched articles	HMA	Number of high-matched articles/NA	Inspired by Etzkowitz and Leydesdorff (11)	1.1 × 10^−2^	0.0	5.9 × 10^−2^

# Number of highly cited articles from Web of Science. Selected from the most recent 10 years of data, highly cited articles reflect the top 1% of papers by field and publication year. The highly cited articles help identify breakthrough research within a research field and are used within the Web of Science to identify and refine the most influential research articles.

* Number of hot articles from Web of Science: Selected by being cited among the top one-tenth of 1% (0.1%) in a current bimonthly period. Articles are selected in each of the 22 fields of science and must be published within the last 2 years.

We applied the term frequency–inverse document frequency (TF-IDF) algorithm (20–22) and cosine similarity to match publication abstracts and patent documents by assessing text similarity. For each hospital, we built one TF-IDF library for publications and one for patents and then calculated two text similarity matrixes based on each TF-IDF library. The two matrices were averaged, and if the text similarity between a publication's abstract and a patent document was >0.36 (17), the article and the patent were regarded to be matched. Then, the number of publication–patent matches in each hospital was recorded. We designed two indicators which are as follows: the number of patents matched per article (NPMA) and the number of articles matched per patent (NAMP). We also identified articles with the top 5% number of matches across all articles of all hospitals (similar to highly matched patents) and developed additional two indicators, namely the number of highly matching articles (HMA) and the number of highly matching patents (HMP). As our aim was to evaluate hospital scientific innovation performance, we developed indicators reflecting relevance between articles and patents, and we did not include any indicator reflecting outcomes or outputs of medical services and the scale and operation of hospitals, such as the survival rate.

### 2.2. The hospital innovation performance evaluation system construction

**Figure 1:**
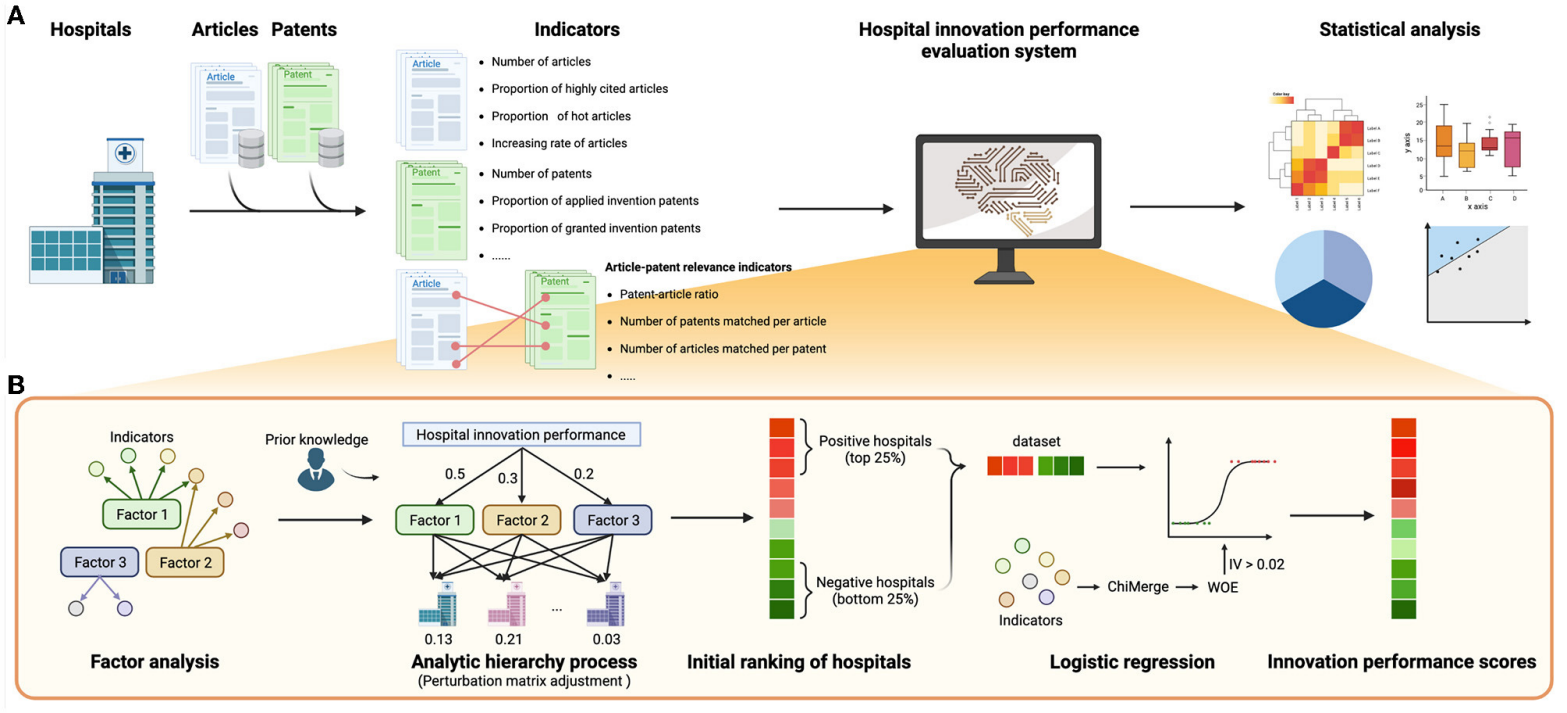
Workflow of hospital innovation performance evaluation system. **(A)** The workflow of this study. **(B)** The steps of the construction of the evaluation system. This illustration was drawn by BioRender (https://www.biorender.com/).

We constructed an evaluation system of healthcare organizations' innovation performance based on Sci-tech relevance (Figure 1).

#### 2.2.1. Step 1. Conduct factor analysis and analytic hierarchy process

Factor analysis and analytic hierarchy process were used to simplify the indicators and complex multi-objective problems (23–25). Before factor analysis, the Kaiser–Meyer–Olkin (KMO) test and the Bartlett test were performed (26, 27), and an indicator with a KMO score >0.5 (28) was included in the factor analysis. The factor with eigenvalues >1.0 (Kaiser rule) (29) and to the left of the “elbow” point in the screen plot (30) was retained. The interpretations of these factors were based on the loading matrix of factor analysis and prior knowledge.

We further performed an analytic hierarchy process (AHP) to determine the weights of the hospital innovation performance factors. We first manually assigned initial weights for all factors of factor analysis based on our prior knowledge. One factor would be assigned greater initial weight if it reflected the publication–patent relevance better. Based on initial weights, a relative importance matrix of factors was created and then the matrix was mapped to the judgment matrix according to the AHP importance scale. If the consistency ratio (CR) was <0.1 (31), it would be assumed that the judgment matrix was qualified for the consistency test, and the values in the eigenvector corresponding to the maximum eigenvalue of the normalized judgment matrix were used as the weights of factors. In addition, to avoid re-assigning initial weights in the case of the failure of the consistency test, we introduced a perturbation matrix (32) to automatically adjust the judgment matrix repeatedly until it passed the consistency test.

#### 2.2.2. Step 2. Fit the function of the innovation performance by logistic regression

The initial ranking of hospitals was obtained by the weighted sum of factors. Although this ranking was based on our prior knowledge, we hypothesized that the top 25% of hospitals in the ranking had better innovation performances than the hospitals in the bottom 25%. Thus, these hospitals were used as positive and negative samples to form a discovery dataset to further optimize the hospital innovation performance ranking.

In this study, logistic regression was performed based on the discovery dataset and 18 original indicators to construct the hospital innovation performance scoring model. In order to reduce the fitting difficulty and to increase model robustness, we introduced ChiMerge (33–35), weights of evidence (WOE) (36, 37), and indicator screening based on information value (IV) (38). First, ChiMerge was conducted to discretize indicators. Then, WOE coding was performed to assign scores to bins of discretized indicators (Formula 1). The indicators with IV < 0.02 were removed (38). WOE coding was adjusted in this study due to the small sample size as follows.

(1) $$\begin{array}{l} {W_{i}}^{{}^{\prime }}=k\times \left(i+\frac{1-n}{2}\right)+\overset{n}{\underset{i=1}{\sum }}\frac{W_{i}}{n}. \end{array}$$

Here, *W*_*i*_ represents the adjusted WOE value, *W*_*i*_ represents the original WOE value of the *i*-th box of an indicator, *k* represents the slope of original WOE values, and *n* represents the number of boxes of the indicator.

Finally, logistic regression analysis was carried out by the scikit-learn package in Python (Formula 2) (39, 40).

(2) $$\begin{array}{l} P\left(y=1\right)=\frac{1}{1+e^{-\theta \times \text{x}}}. \end{array}$$

Here, θ is the parameter of the logistic regression, ***x*** is the adjusted WOE values of indicators of hospitals, and y is the label indicating whether each sample is positive or not. We added the “squared magnitude” of the coefficient as the penalty term (L2 regularization) to control the effect of the collinearity of indicators. The receiver operating characteristic curve (ROC) of 5-fold cross-validation was plotted to examine the reliability of the model (41, 42). Different from the conventional machine learning process, the goal of logistic regression here was not to build a prediction model of hospital innovation scores but to optimize the weights of original indicators and build an innovation scoring function based on existing initial innovation rankings. The coefficient ***θ*** of logistic regression was min-max normalized to the interval of (1, 2) to form ***θ′***, and the weighted sums of ***θ′*** and adjusted WOEs were the innovation performance scores *S* of sample hospitals (Formula 3). Then, this study sorted all the hospitals according to innovation performance scores to obtain the hospital innovation performance rankings.

(3) $$\begin{array}{l} S=\theta \prime \times \;W\prime \end{array}$$

#### 2.2.3. Step 3. Design regional innovation performance index (RIP) of healthcare organizations

We developed an index, namely the regional innovation performance index (RIP), to estimate innovation performance on the province level. We calculated the RIPs in 31 regions in China according to the scores and locations of sample hospitals (Formula 4).

(4) $$\begin{array}{l} R=\overset{n}{\underset{i=1}{\sum }}\left(s-r_{i}\right) \end{array}$$

Here, *R* is the RIP of a region, *n* is the number of hospitals in the region, *s* is the number of hospitals in all the regions, and *r*_*i*_ refers to the innovation performance rankings of the *i*-th hospital in a certain region. We did not normalize RIP with the number of hospitals in each region because we aimed to construct RIP to estimate the aggregation of regional medical innovation performance instead of the average performance of hospitals.

#### 2.2.4. Step 4. Analyze hospital innovation performance in the digital health field

To identify the relevant publications and patents of digital health across hospitals, we used 24 keywords to build the search terms, including “medication reminder app,” “smart drug,” and “Digital health application” (Supplementary Table S3). The indicators of digital medicine were constructed, and the hospital innovation performance score of digital health was calculated according to the same process mentioned above. Mediation analysis, which is a commonly used statistical analysis method to determine the indirect relationships between the variables, was applied to determine the causal relationship between digital health and hospital rankings. The variables were normalized through the StandardAero function in the scikit-learn (40) package. Then, mediation analysis was performed by mediation package with 1,000 bootstraps for significance testing (43).

### 2.3. Statistical analysis

When not specified otherwise, the statistical analyses have been performed with Python (version 3.6.0) or R (version 4.1.1). Differences were considered statistically significant when *P*-value was < 0.05. The relationship between different indicators was assessed by Spearman's correlation analysis where appropriate.

## 3. Results

### 3.1. Validity of the evaluation model for hospital innovation performance

In this study, 159 sample hospitals were included (Supplementary Table S1), 692,342 publications and 45,106 patents were attributed to these hospitals, and 18 indicators were constructed (Supplementary Table S4). After the KMO test (KMO = 0.598) and the Bartlett test (*P* < 0.01), factor analysis was performed and five factors were obtained by the Kaiser rule and scree test (Supplementary Figure S1, Supplementary Table S5). According to factor loading from the factor analysis (Supplementary Table S6) and prior knowledge, initial weights of five factors were assigned (Supplementary Table S5). Among them, factor 1 showed high loading from NPMA (loading = 0.970) and HMA (loading = 0.937), which indicated the strong Sci-tech relevance stimulated patent applications. Factor 1 was assigned the highest initial weight of 35% to highlight the positive correlation between scientific research and technological innovation. PGIP (loading = 1.011) and PUP (loading = −0.813) showed strong loading to factor 2 which illustrated the capacity of technology application, with an initial weight of 25%. Factor 3 contained some Sci-tech relevance indicators and represented the technology innovation performance of hospitals, with a weight of 25%. In addition, factor 4 and factor 5 reflected the scientific research capacity of hospitals, with a weight of 10%and 5%, respectively.

Through ChiMerge (maximum number of boxes was 5, and confidence was 0.99) and weight of evidence (WOE) coding, NP and PAR were removed in IV-based indicator screening (IV < 0.02), and then logistic regression was performed (Supplementary Table S4). As far as there is no existing hospital ranking based on innovation performance, which is our goal, there is no golden dataset for model validation. Instead, we validated the model with 5-fold cross-validation. The 5-fold cross-validation ROC curve showed that the average area under the curve (AUC) was 0.99, proving the validity of the logistic regression model (Supplementary Figure S2). Finally, the innovation performance of all hospitals was scored based on the logistic regression model (likelihood ratio test, *P* < 0.01, Supplementary Table S6).

### 3.2. Analysis of factors influencing hospital innovation performance

**Figure 2:**
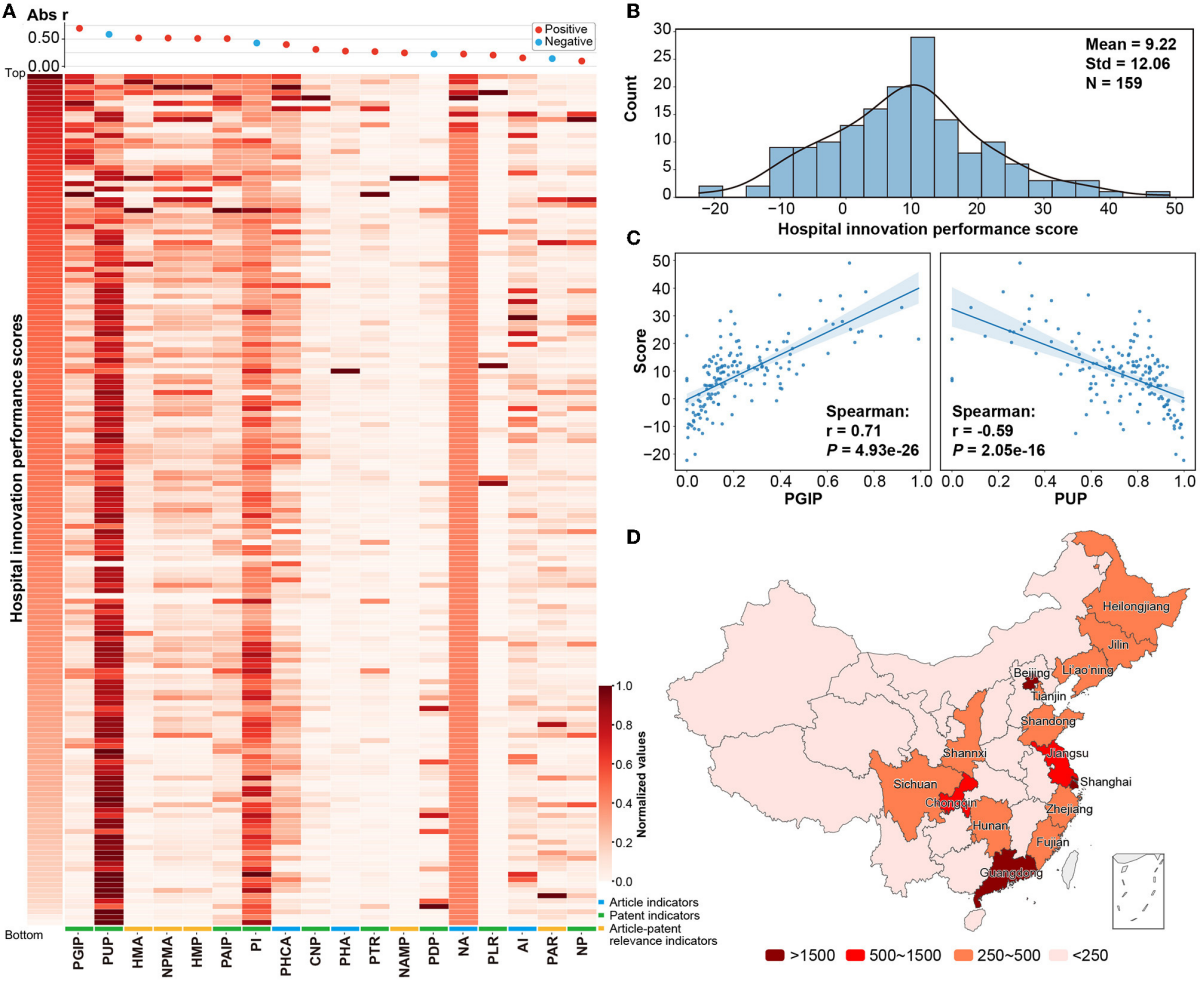
Distribution of hospital innovation performance indicators. **(A)** A heatmap plot between the hospital innovation performance score and the indicators was constructed. The hospital score was on the vertical axis, and 16 indicators enrolled in the logistic regression analysis were on the horizontal axis. All the values were normalized to the interval of [0, 1]. **(B)** The distribution of hospital innovation performance scores. **(C)** The distribution of the proportion of granted invention patents (PGIP) and proportion of utility model patents (PUP) and the correlation with hospital innovation performance scores. **(D)** A distribution of RIPs on regions was mapped. Regions whose data were not included in the study were marked in white color, including Hong Kong, Macao, and Taiwan. Detailed results are shown in Supplementary Table S2.

The innovation performance score of 159 hospitals showed a normal distribution with a mean value of 9.22 and a standard deviation of 12.06 (Figure 2B). In this evaluation system, the patent indicators and Sci-tech relevance indicators had a high weight and strong correlation with hospital innovation performance scores (Figure 2A, Supplementary Table S6). Among them, PGIP (*r* = 0.71, *P* = 4.93 × 10^−26^) and PUP (*r* = −0.59, *P* = 2.05 × 10^−16^) were significantly positively and negatively correlated with hospital innovation performance scores, respectively (Figure 2C), which indicated that more granted invention patents were conducive to hospital innovation performance, while utility model patents were the opposite. Indicators of HMP (*r* = 0.56, *P* = 1.77 × 10^−14^), NAMP (*r* = 0.64, *P* = 1.25 × 10^−19^), and NPMA (*r* = 0.48, *P* = 1.21 × 10^−10^), reflecting the Sci-tech relevance, showed a strong positive correlation with hospital innovation performance score (Figure 2A). In addition, article indicators were also positively correlated with hospital innovation performance score, but the correlation was weak (*r* < 0.50, Figure 2A).

**Figure 3:**
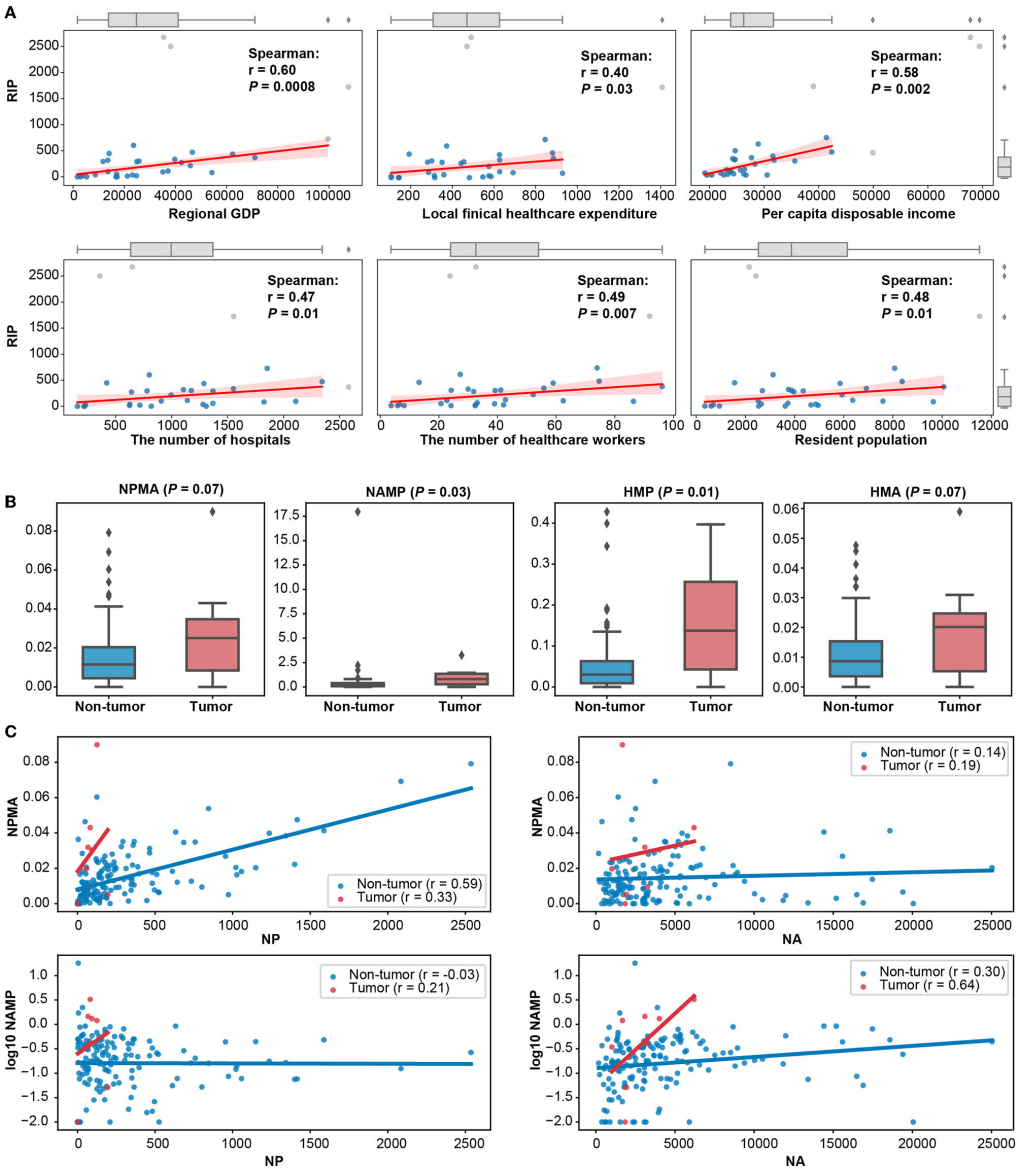
Different factors affecting hospital innovation performance. **(A)** Scatter plots and fitting curves among six regional statistics and RIP. Outliers were detected by 1.5 times the interquartile range and marked with gray dots. **(B)** Box plots of publication–patent relevance indicators of cancer and non-cancer hospitals. **(C)** Scatter plot of publication–patent relevance indicators and NA or NP in cancer and non-cancer hospitals. Spearman's coefficients and *p*-value were marked in the plots.

The development of hospital innovation levels in different regions of China was unbalanced (Figure 2D, Supplementary Table S2). The RIPs of Beijing, Shanghai, Guangdong, Jiangsu, and Chongqing were higher than 1,500, while RIPs were generally lower in most inland regions, except for the districts of Chongqing, Sichuan, Shanxi, and Hunan. This situation may be related to the levels of regional economic development and population (Figure 3A). There was a positive correlation between RIP and the regional GDP (*r* = 0.60, *P* < 0.01). The results also indicated that there was a positive correlation between RIP and per-capita disposable income (*r* = 0.58, *P* < 0.01). On the other hand, local healthcare expenditure (*r* = 0.40, *P* = 0.03), the number of hospitals (*r* = 0.47, *P* = 0.01), the number of healthcare workers (*r* = 0.49, *P* < 0.01), and the residential population size (*r* = 0.48, *P* = 0.01) showed limited relevance with RIP (Figure 3A).

In addition, there were disparities in Sci-tech relevance among different types of hospitals (Figure 3B). Compared with non-cancer hospitals, the Sci-tech relevance indicators, NPMA (*P* = 0.07), NAMP (*P* = 0.03), HMP (*P* = 0.01), and HMA (*P* = 0.07), were significantly higher in cancer hospitals (Figure 3B). NPMA had a stronger trend of positive correlation with NP in non-cancer hospitals (*r* = 0.59), while NAMP had a stronger trend of positive association with NA in cancer hospitals (*r* = 0.64, Figure 3C).

### 3.3. Comparison with existing ranking systems

The hospital innovation performance ranking was different from other Chinese hospital ranking systems (Spearman's correlation coefficient, Supplementary Figure S3, Supplementary Table S4), such as Chinese hospital competitiveness rankings, Chinese hospital science and technology value rankings, Chinese best hospital rankings, and Chinese hospital Natural Index rankings, showing unique characteristics of hospitals. In the hospitals ranked differently from other rankings (Supplementary Table S7), hospitals with high rankings in our evaluation model had greater scores in factor 1 and factor 2. It indicated that our hospital evaluation model emphasized Sci-tech relevance, which was usually neglected by other hospital assessment systems.

### 3.4. Impact of digital health on hospital innovation performance

**Figure 4:**
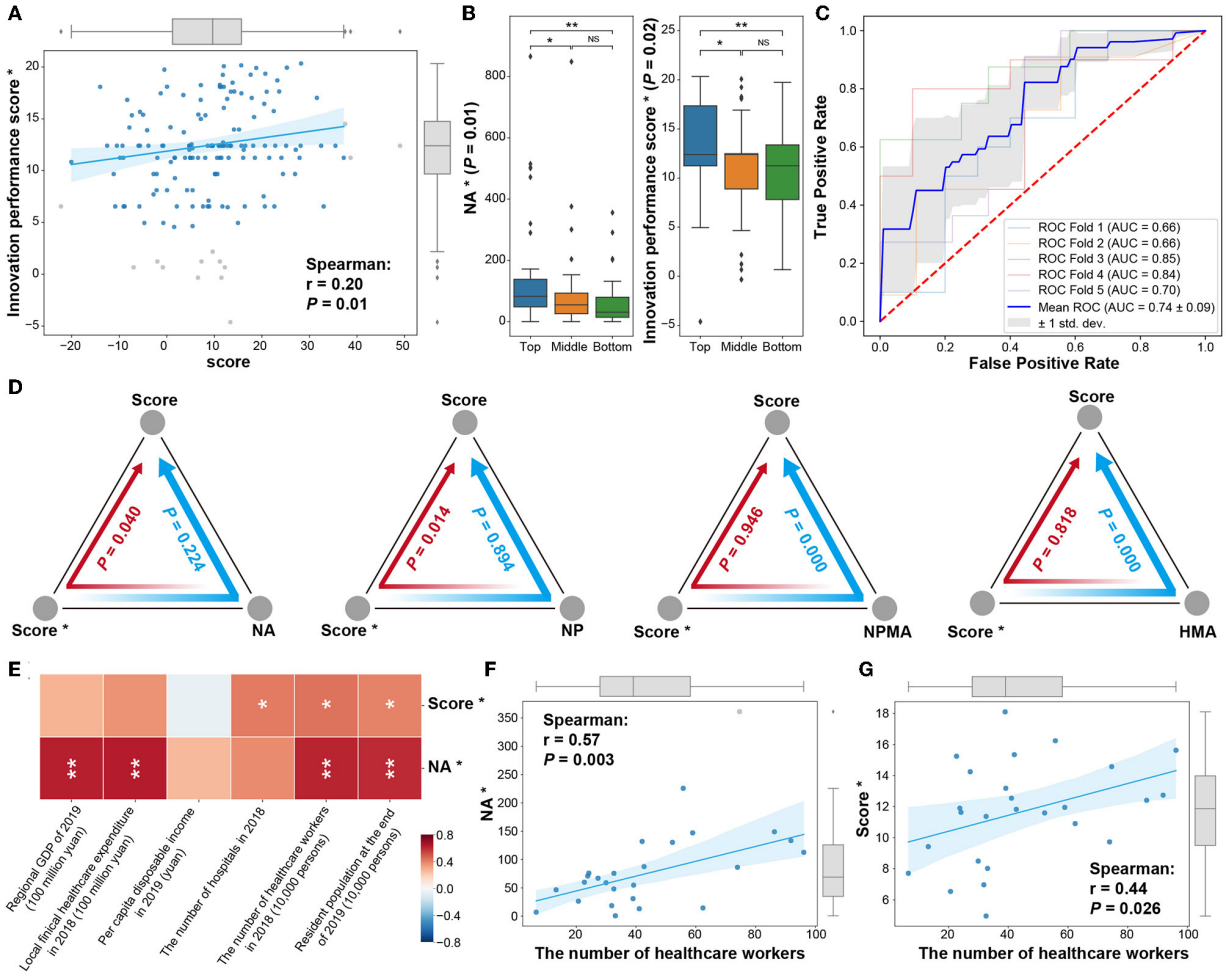
Hospital digital health innovation performance. **(A)** Correlation analysis between digital health performance score* and overall score. **(B)** Distribution of digital health-related indicators (NA*, score*) of the top 50 (Top), bottom 50 (Bottom), and other hospitals in the middle of the overall hospital ranking. **(C)** The ability of digital medical indicators to distinguish between the top 50 (Top) and bottom 50 (Bottom) hospitals in overall ranking. **(D)** The mediation effect of digital health hospital innovation performance score (score*) and overall hospital NA, NP, NPMA, and HMA indicators on the overall hospital innovation performance score. **(E)** The relationship between digital health NA*, score*, and regional economic and other indicators. **(F)** The relationship between the number of healthcare workers and NA*. **(G)** The relationship between the number of healthcare workers and score* (*: digital health-related indicators; outliers were detected by 1.5 times the interquartile range and marked with gray dots.). In the boxplot and heatmap, *, *P* < 0.05, **, *P* < 0.01, NS, not significant.

Extracting digital health-related articles and patents, we conducted the same assessment of hospitals' performance on innovation in digital health (Supplementary Table S8). Among them, there was a significant positive correlation between digital health innovation performance scores and overall hospital innovation performance scores (*r* = 0.20, *P* = 0.01, Figure 4A). The NA^*^ and score^*^ of the top 50 hospitals on digital health-related indicators were significantly higher than those of the bottom 50 hospitals (*P* < 0.01, Figure 4B). The top 50 and bottom 50 hospitals in the overall innovation ranking could be distinguished by the digital health-related indicators (mean AUC = 0.74, Figure 4C).

The digital health-related indicators, such as NA^*^, CNP^*^, and score^*^, were related to overall indicators (Supplementary Figure S4). Then, the mediation analysis was performed to determine the causal relationship between digital health and hospital innovation performance (Figure 4D). The hospitals' digital health innovation performance was stimulated by the overall increase of articles (*P* = 0.04) and patents (*P* = 0.01), thus improving the hospital's overall innovation ranking (Figure 4D). In addition, the hospitals' digital health innovation performance affected the hospital's overall innovation score with the mediation of Sci-tech relevance indicators (NPMA and HMA, *P* < 0.01, Figure 4D).

The level of digital health in hospitals was also affected by regional development (Figure 4E). Interestingly, the hospitals' digital health innovation performance score was more significantly related to the number of healthcare workers and the resident population (*P* < 0.05, Figures 4E–G).

## 4. Discussion

The evaluation model in this study can be broadly applied. First, the perturbation matrix minimizes the manual workload of re-assigning weight to common factors in the approach of automatically adjusting the weights of indicators in AHP when the consistency test fails. Second, the measurements of chi-square, WOE coding, and IV value are implemented in this research to reduce the complexity of the raw data and the difficulty of model fitting in the process of data discretization, coding, and indicator selection, while retaining as much original data distribution feature contained in the data as possible. Third, the model is constructed by logistic regression, which has the advantages of low model complexity, low training cost, high robustness, and only needs a small number of hyperparameters. Finally, this model did not include any subjective data, such as peer appraisals. This means that the results were completely the reflection of hospital innovation performance with the highlight of Sci-tech relevance.

Scientific research and technological innovation in oncology are more mutually supportive than in non-oncology areas. Most of the medical innovation products in the field of oncology are anti-tumor drugs, and the median cost of a clinical trial for an innovative drug is $33.4 million (44). In terms of time spent, the average drug development cycle is at least 13.5 years, in which, the average time spent on a clinical trial is 8 years (45). Due to the huge costs and the fierce competition in this field, hospitals, specializing in oncology areas, will establish a comprehensive patent protection system to protect the legal rights of their innovations generated by basic research to guarantee their future high revenue when their patented technology is transferred, so the correlation between publications and patents in this disease field is obviously better than in other fields. It is worth noting that because the size of non-cancer hospitals was small, the results here need to be treated with caution.

The regions with high RIP were concentrated on the eastern coastal areas with a higher level of economic development. These regions tended to have better local medical innovation performance. The population's demand for advanced healthcare was one of the main external drivers for hospitals to pay high attention to the synergy between technology innovation and academic research. Therefore, the promotion of scientific and technological innovation policies could be carried out in advance in areas with better economic development. On the other hand, increasing the hospitals' size was an ineffective approach to improving their innovative capacity.

Our evaluation system of hospitals in China has no relevance to Chinese Hospitals' Competitiveness Rankings because of the different metrics applied to both rankings. Chinese Hospitals' Competitiveness Rankings evaluate the academic capability of hospitals by the index of the number of National Science Foundation projects, national key laboratories, and national key disciplines and academicians but exclude papers and patents (10). Chinese Hospital Science and Technology Evaluation Metrics, China's Hospital Rankings, and Nature Index contain the metrics of papers or patents, so the result of this study is related to these evaluated systems. However, this study innovatively constructed the indexes of the number of patents matched per article, the number of articles matched per patent, the proportion of highly matched patents, and the proportion of highly matched articles in building the evaluation system and gave more significance to highlight the advantages of the model in evaluating the differences in the correlation between scientific research and technological innovation among hospitals, which was also ignored by the three rankings. The metrics and their weights of this study are also dissimilar to the three ranking systems, so the result is weakly correlated to them.

Healthcare workers' innovations in the digital health field enhance the Sci-tech relevance and benefit the innovation performance in hospitals. The study also implies that the development of digital health in hospitals depends on the input of medical personnel.

## 5. Conclusion

In the digital health era, the combination of science and technology is more evident in the innovation behavior of healthcare organizations. The evaluation system of medical institutions is a pioneer for healthcare workers' innovation behavior. However, existing ranking systems of healthcare organizations related to innovation performance traditionally paid attention to patents and articles independently. The novelty of this study is designing the HMA, HMP, NAMP, and NPMA indicators based on publications and patents data of sample hospitals, reflecting the Sci-tech relevance, and these metrics showed a strong positive correlation with hospital innovation performance. This assessment model evaluates the innovation behavior of healthcare organizations from the perspective of scientific and technological relevance, which is more in line with the behavioral characteristics of healthcare organizations' innovation in the digital health era, and provides a new perspective of knowledge transfer for policymakers to more accurately judge the innovation strength of healthcare organizations.

## Data availability statement

The original contributions presented in the study are included in the article/Supplementary material, further inquiries can be directed to the corresponding authors.

## Author contributions

WG and LS contributed to the formal analysis and writing—original draft. JW contributed to the formal analysis. LS and WC contributed to data curation, data validation, and methodology. ZA contributed to writing—reviewing and editing. DW contributed to resources and writing—reviewing and editing. JL contributed to conceptualization, funding acquisition, and writing—original draft, reviewing, and editing. All authors read and approved the final manuscript.
