# Supplementary figures and images for: Evaluation of Chinese healthcare organizations' innovative performance in the digital health era
Source: Front Public Health. 2023 Jul 6;11:1141757. doi: 10.3389/fpubh.2023.1141757 (PMC10359909; doi:10.3389/fpubh.2023.1141757)

# scree test

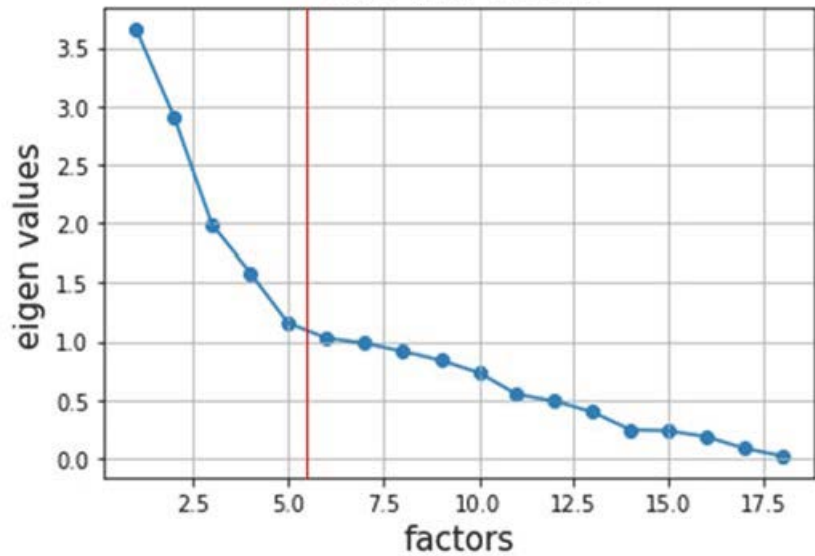

Supplement: Supplementary file 2 [file Image_1.pdf]

Receiver operating characteristic example

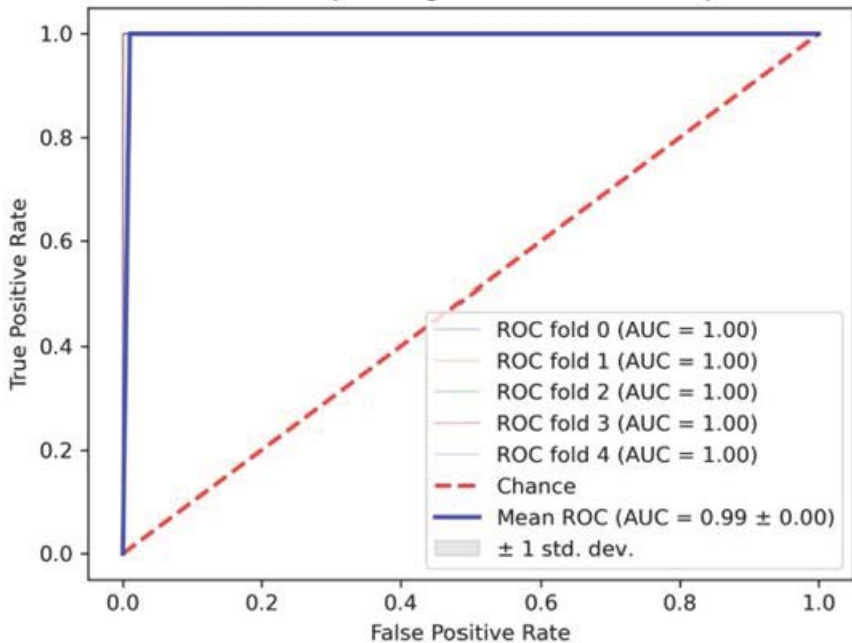

Supplement: Supplementary file 3 [file Image_2.pdf]

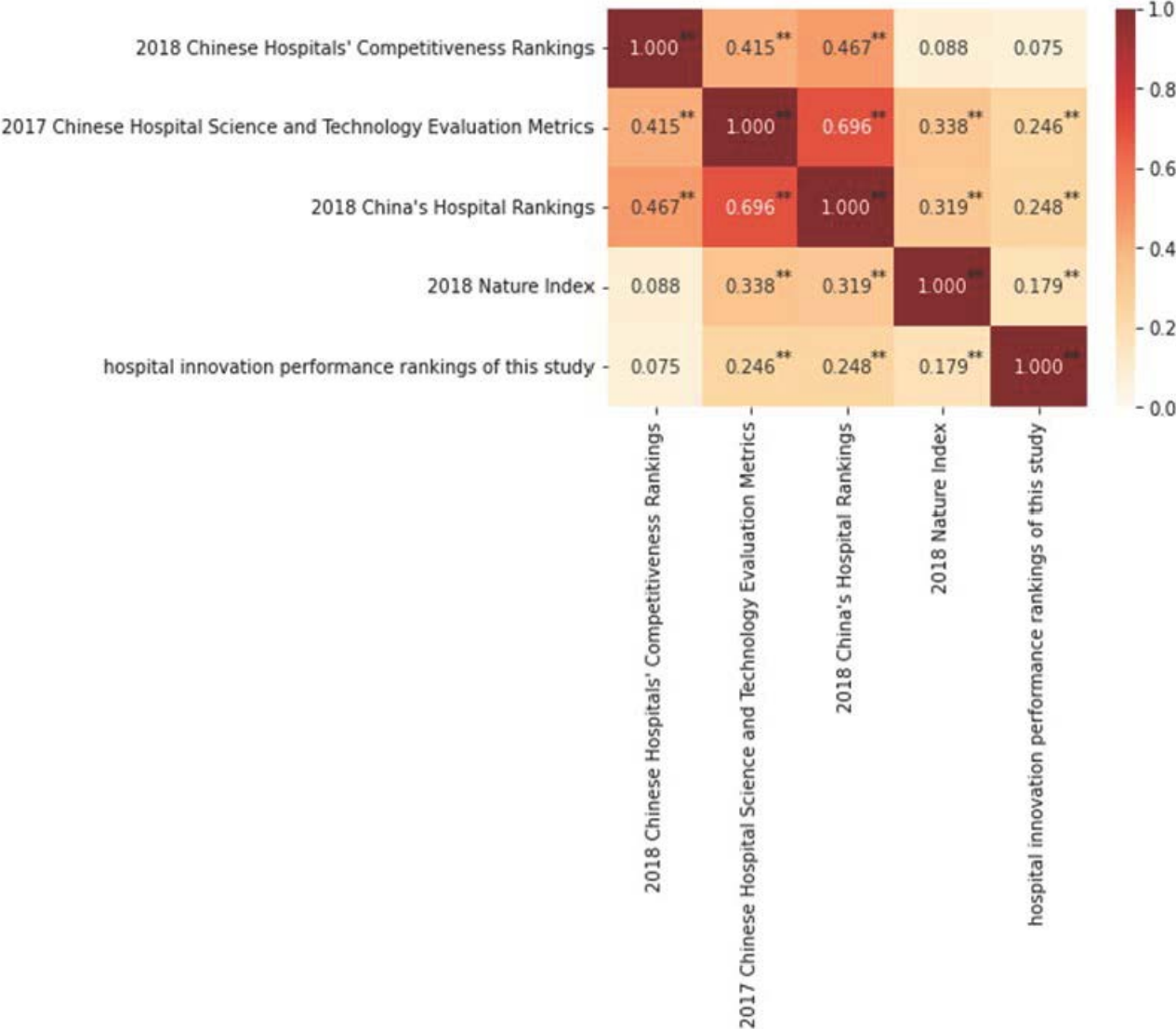

Supplement: Supplementary file 4 [file Image_3.pdf]

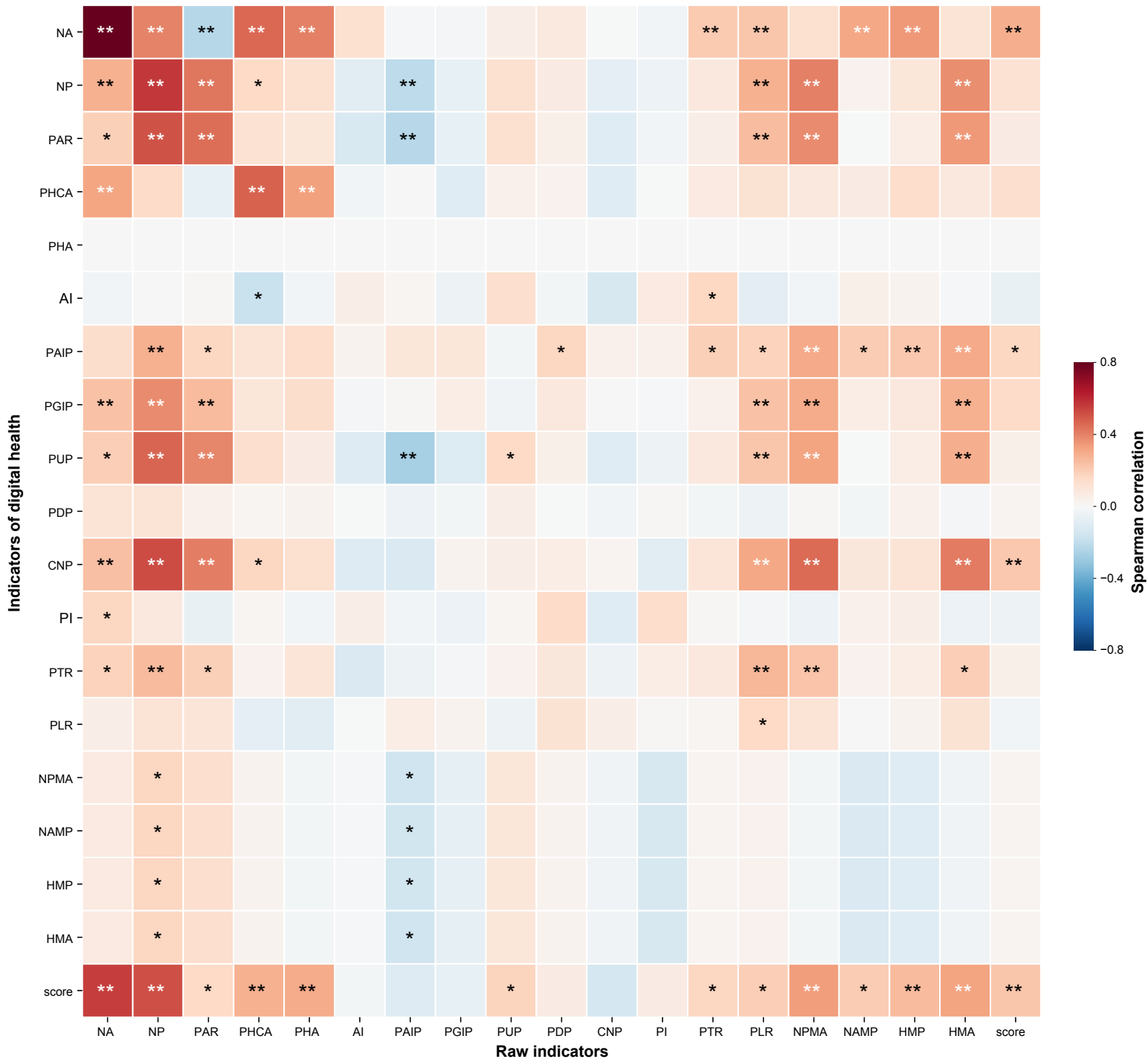

Supplement: Supplementary file 5 [file Image_4.pdf]
